# LINC00174 Promotes Colon Cancer Progression by Regulating Inflammation and Glycolysis by Targeting the MicroRNA-2467-3p/Enolase 3 Axis

**DOI:** 10.1155/2023/8052579

**Published:** 2023-07-05

**Authors:** Sheng Xu, Jiawei Lin, Rong Chen, Junjie Xie, Enquan Yuan, Fajie Cen, Fanbiao Kong

**Affiliations:** ^1^Department of General Surgery, People's Hospital of Guangxi Zhuang Autonomous Region, 530021 Nanning, China; ^2^Oncology Department, General Hospital of Central Theater Command, Wuluo 627, Wuhan, 430070 Hubei Province, China

## Abstract

**Objective:**

To elucidate the mechanism by which LINC00174 promotes colon cancer progression by targeting the microRNA-2467-3p (miR-2467-3p)/enolase 3 (ENO3) axis to regulate inflammation and glycolysis.

**Methods:**

The expression of LINC00174 and ENO3 in colon cancer tissues, its relationship with survival rate, and correlation were analyzed using bioinformatic analysis. The effects of LINC00174 overexpression and silencing on the biological behavior of and inflammation in colon cancer cells were analyzed via transfection experiments. The target relationships between miR-2467-3p or LINC00174 and ENO3 were verified using sequence prediction and the dual-luciferase reporter assay, respectively. Furthermore, LINC00174- and/or miR-2467-3p-overexpressing cells were prepared to determine the effects on ENO3 protein levels and glycolysis. Finally, the effects of LINC00174 and/or miR-2467-3p overexpression on colon cancer, ENO3 protein levels, and inflammation were analyzed using a tumor-bearing mice model.

**Results:**

LINC00174 and ENO3 were overexpressed and associated with a lower survival rate. LINC00174 was positively correlated with ENO3 in colon cancer tissues. Furthermore, the overexpression of LINC00174 in colon cancer cell lines promoted the proliferation, migration, and invasion of colon cancer cells and inflammation but inhibited apoptosis. The overexpression of miR-2467-3p inhibited ENO3 protein levels, which was attenuated via LINC00174 overexpression. Furthermore, it inhibited the biological behavior of and inflammation and glycolysis in colon cancer cells and blocked their LINC00174-induced promotion. Moreover, using animal experiments, the regulatory effects of LINC00174 on tumor growth, ENO3 protein levels, and inflammation via miR-2467-3p were confirmed.

**Conclusion:**

LINC00174 promotes the glycolysis, inflammation, proliferation, migration, and invasion of colon cancer cells and inhibits apoptosis. The cancer-promoting mechanism of LINC00174 is related to targeting miR-2467-3p to promote ENO3 protein levels.

## 1. Introduction

A global statistical report revealed 1,148,515 new cases of colon cancer and 576,858 colon cancer-related deaths in 2020, accounting for 6.0% and 5.8% of all tumors, respectively; this makes colon cancer the fifth most common cancer type [1]. Surgery or combination chemotherapy is the main treatment strategy to decrease tumor burden and prolong patient survival [2, 3]. Nevertheless, patients with colon cancer have a poor prognosis, with an overall 5-year survival rate of approximately 60% [4, 5]. However, the detection of this cancer type is difficult at an early stage, and the survival rate of advanced colon cancer is only 8%–30% [6, 7]. Colon cancer is also associated with chronic inflammation, and diseases such as ulcerative colitis may further progress to colon cancer [8, 9]. Furthermore, chronic inflammation is conducive to tumor cell metastasis [10, 11]. Moreover, increased interleukin- (IL-) 1*β* and IL-8 levels promote the migration and invasion of colon cancer cells [12, 13]. Therefore, it is extremely crucial to determine colon cancer pathogenesis.

Long noncoding RNAs (lncRNAs) can competitively adsorb microRNAs (miRNAs) and inhibit their posttranscriptional gene expression [14]. Furthermore, they can promote colon cancer progression by inducing inflammation, such as taurine upregulated 1 [15, 16]. LINC00174 has been recently discovered as a cancer-promoting lncRNA; it plays a role in promoting the progression of liver cancer [17], breast cancer [18], and osteosarcoma [19]. A sequencing-based study reported that LINC00174 is associated with colon cancer prognosis and is involved in regulating glucose metabolism [20]. However, the effect and mechanism of LINC00174 on the biological behavior of colon cancer cells remain unclear.

Tumor cells produce ATP mainly via glycolysis, even if the oxygen supply is sufficient; this is known as the Warburg effect [21, 22]. Tumor cells can rapidly obtain energy via glycolysis, which is conducive to tumor cell growth [23]. The Warburg effect can increase the aggressiveness of colon cancer cells; therefore, reversing glycolysis can be a potential approach to treat colon cancer [24, 25]. Enolase 3 (ENO3) is a *β*-enolase that regulates striated muscle development [26]. Genomic analysis revealed that ENO3 is associated with the risk of colon cancer and that it is involved in the regulation of glycolysis [27]. Elevated levels of glycolysis result in the activation of inflammatory pathways [28], which are also involved in tumor progression [29]. However, this mechanism remains unvalidated at the cellular and animal levels, and the mechanism by which ENO3 expression is regulated remains unclear.

The present study is aimed at determining the mechanism by which LINC00174 regulates ENO3, glycolysis, and inflammation and elucidates its effects on colon cancer. Our goal is to provide a novel theoretical foundation for colon cancer diagnosis and treatment.

## 2. Materials and Methods

### 2.1. Bioinformatic Analysis

Colon cancer cases in The Cancer Genome Atlas (TCGA) database were analyzed using the starBase tool. For differential analysis, 471 tumor tissue samples and 41 normal tissue samples were included. For survival analysis, 447 samples were included. The survival rate of the LINC00174 or ENO3 high- and low-expression groups was analyzed and compared using the logrank test. Further, the correlation between LINC00174 and ENO3 in colon tissues was analyzed using the Pearson test.

### 2.2. Cell Culture

FHC, a human colon epithelial cell line (CRL-1831); Caco-2, a colon cancer cell line (HTB-37); SW480 (CCL-228); COLO201 (CCL-224); and HCT116 (CCL-247EMT) were acquired from American Type Culture Collection (Manassas, VA, USA). All cell lines were maintained in Dulbecco's modified Eagle medium (DMEM) (Gibco, USA) supplemented with 10% fetal bovine serum (FBS), 100 mg/mL streptomycin, and 100 units/mL penicillin (Sigma-Aldrich, USA). The cells were cultured in a 5% CO_2_ incubator at 37°C and 95% humidity. In some experiments, the cells were treated with 5 mM 2-deoxy-d-glucose (2-DG) for 48 h to inhibit cellular glycolysis.

### 2.3. Cell Transfection

Transfection experiments to silence LINC00174 were performed using Caco-2 cells with relatively high LINC00174 expression. Transfection experiments to overexpress LINC00174 were performed using COLO201 cells. Moreover, Caco-2 cells were used to cotransfect LINC00174 and the miR-2467-3p mimic. siLINC00174, siENO3, LINC00174, miR-2467-3p mimic, and corresponding siNC/NC/mimic NC were obtained from GenePharma Co., Ltd. (China). Briefly, 50 pmol (0.67 *μ*g) plasmid was diluted with 25 *μ*L of serum-free DMEM (reagent A). Then, 1 *μ*L of Entranster™-R4000 (Engreen) and 24 *μ*L of serum-free DMEM were mixed for 25 min (reagent B). The transfection complex was prepared by thoroughly mixing 25 *μ*L of reagent A and 25 *μ*L of reagent B (aspirate 10 times using a pipette) and standing for 15 min. Cells in 0.45 mL of complete medium were transfected with 50 *μ*L of the transfection complex. siNC/NC/mimic NC plasmids were used as controls.

### 2.4. RT-qPCR

For analysis, 400 *μ*L of TRIzol (Thermo Fisher, USA) was added to the cells or tissues in a 1.5 mL sterile RNase-free EP tube. The cells or tissues were ground to a homogenate, followed by the addition of 600 *μ*L of TRIzol and mixing for 5 min. Then, the cells were centrifuged for 5 min (4°C, 12,000 × g), and the pellet was discarded. TRIzol and chloroform were mixed at a ratio of 1 : 2 and added to the supernatant of the previous step, followed by shaking for 15 s. Thereafter, isopropanol was added to precipitate the RNA in the aqueous phase. The collected RNA pellet was carefully washed with 75% ethanol to remove impurities. RNA was redissolved in diethylpyrocarbonate-treated water and stored at −80°C. RNA was reverse transcribed into cDNA using the PrimeScript RT reagent kit (RR047A, Takara) under the following conditions: 37°C/15 min and 98°C/5 min. qPCR amplification was performed using the SYBR Green reagent (Takara, Japan) under the following conditions: 94°C for 3 min, followed by 40 cycles at 94°C for 15 s, 58°C for 20 s, and 72°C for 30 s. The expression of LINC00174 and mRNAs was normalized to that of GAPDH using the 2*^−ΔΔCt^* method. The expression of miR-2467-3p, miR-3918, and miR-542-3p was normalized to that of U6. The primers used were as follows: LINC00174 F: 5′-AAGCCCCTGGGGAATGTTTC-3′, R: 5′-AAGCCCCTGGGGAATGTTTC-3′; IL-1*β* F: 5′-TCCTTGCCCTTCCATGAACC-3′, R: 5′-TTACTTGGCACCCTGTTTGC-3′; IL-8 F: 5′-GGCAGCCTTCCTGATTTCTG-3′, R: 5′-AATTTCTGTGTTGGCGCAGTG-3′; IL-10 F: 5′-GAGATGCCTTCAGCAGAGTGA-3′, R: 5′-ACTCATGGCTTTGTAGATGCCT-3′; GAPDH F: 5′-TTTCTGACTCCGTGAACCGC-3′, R: 5′-AGTCCTTCCACGATACCAAAGTT-3′; miR-2467-3p F: 5′-CCTGAGGCTCTGTTAGCCTT-3′, R: 5′-ACAGACCCTGAGCCTCTC-3′; miR-3918 F: 5′-GGTTAAGCCATGGGACAGGG-3′, R: 5′-CTACGAACCAGGTGCAGGG-3′; miR-542-3p F: 5′-TCGGGGATCATCATGTCACG-3′, R: 5′-GAGTGGCTCCCAGACCTTTC-3′; and U6 F: 5′-CTCGCTTCGGCAGCACA-3′, R: 5′-AACGCTTCACGAATTTGCGT-3′.

### 2.5. Measurement of IL-1*β*, IL-8, and IL-10 Levels via Enzyme-Linked Immunosorbent Assay (ELISA)

In transfection experiments, Caco-2 cells with relatively high LINC00174 expression were used to silence LINC00174, whereas COLO201 cells were used to overexpress LINC00174. The cells were collocated and centrifuged at 1000 × g for 15 min at 4°C. The supernatant was collected and transferred into 96-well plates. The levels of IL-1*β*, IL-8, and IL-10 were measured using ELISA kits (Invitrogen, USA) following the manufacturer's instructions. Optical density (OD) was measured using a microplate reader (Biotek, Winooski, VT, USA). The levels of IL-1*β*, IL-8, and IL-10 were expressed as microgram per milliliter.

### 2.6. Cell Counting Kit-8 (CCK-8) Assay

The cell suspension (100 *μ*L) at a density of 5 × 10^4^/mL was added into the wells of a 96-well plate. After 24, 48, and 72 h, 10 *μ*L of the CCK-8 solution was added. The plates were gently mixed on an orbital shaker for 1 min at 37°C to ensure uniform mixing. The plates were then incubated for 2 h for the dehydrogenation reaction. The OD was measured at a wavelength of 450 nm using a microplate reader (Biotek).

### 2.7. Flow Cytometry

Cells were collected and trypsinized without EDTA. After washing with PBS and centrifuging the samples two times at 2000 rpm for 5 min, 5 × 10^5^ cells were collected and resuspended in 500 *μ*L of binding buffer. Then, 5 *μ*L of annexin V-fluorescein isothiocyanate and propidium iodide (Sanjiang Biological Technology Co., Ltd., China) was used to mark apoptotic cells (15 min in the dark). After 30 min, flow cytometry (BD FACSCalibur, USA) was performed for detection. Normal cells were used for fluorescence compensation modulation to set the position of the cross gates.

### 2.8. Scratch Healing Assay

Cells were grown to 90%–95% confluency in 6-well plates to form a monolayer under the abovementioned conditions. Then, a 200 *μ*L pipette tip was used to make vertical scratches from top to bottom. This was considered 0 h, and the scratch width was recorded. After the streaked cells were washed away, the cells were cultured in serum-free medium. Photographs were captured after 24 h to assess scratch healing.

### 2.9. Transwell Assay

Matrigel (1 : 8 dilution; Corning, USA) was added to the upper chamber, and the sample was incubated at 37°C for 30 min. Then, 600 *μ*L of complete medium (20% FBS) was added to the lower chamber of the 24-well Transwell device. The cells (5 × 10^4^/mL) were cultured in serum-free medium at 37°C for 24 h for starvation treatment. After digestion, 100 *μ*L of cell solution (5 × 10^4^/mL) was added to the hydrated Transwell chamber. After 24 h, the uninvaded cells were washed away, and the infiltrated cells in the lower chamber were fixed with 95% ethanol and stained with 0.1% crystal violet for 20 min at room temperature (25°C). The number of cells was counted in five random fields in a ×400-fold field of view.

### 2.10. Analysis of Glycolysis Levels

Glycolysis level was determined by detecting glucose intake and lactic acid secretion. Briefly, 1 × 10^5^ cells were seeded in a Petri dish and incubated for 24 h under the abovementioned conditions. Then, the medium was removed, and 5 mL of medium without FBS and L-glutamine was added. After incubation for 8 h, the supernatant was collected via centrifugation. Glucose intake was detected using the Amplex Red Glucose/Glucose Oxidase Assay Kit (Molecular Probes, CA, USA). Lactic acid production was detected using a lactic acid detection kit (BioVision, CA, USA).

### 2.11. Dual-Luciferase Reporter Assay

For the dual-luciferase reporter assay, 1 *μ*g of wild-type (wt) LINC00174/ENO3-pGL4 (Promega Corporation, USA) or mutant LINC00174/ENO3-pGL4 (induced using the QuickMutation™ Kit, Beyotime, China), 50 nmol miR-2467-3p mimic/mimic NC, and 150 ng *Renilla* luciferase plasmid (Beyotime) were transfected into 3 × 10^4^ cells using Lipofectamine® 2000 at 37°C for 36 h. Luciferase activity was measured using the dual-luciferase reporter gene detection kit (Promega Corporation). All data were normalized to *Renilla* luciferase activity.

### 2.12. Western Blotting

The cells or tissues were collected and incubated with RIPA lysis solution on ice for 30 min. Then, total protein was obtained via centrifugation at 12000 × g for 20 min at 4°C. Sodium dodecyl sulfate-polyacrylamide gel electrophoresis was performed to separate the proteins. The rabbit monoclonal primary antibody of ENO3 (1 : 1000) (ab157474, Abcam, USA) was added to the membrane (4°C, overnight). Then, the membrane was washed two times with Tris-buffered saline/0.1% Tween (TBST) solution. The membrane was then incubated with secondary antibodies (1 : 2000, ab6721) for 2 h at 37°C. The membrane was washed three times with TBST. The protein blots were visualized using an ECL kit (Solarbio) and detected using IPP6.0 (Media Cybernetics, USA).

### 2.13. Construction of the Syngeneic Model

Four-week-old male normal BALB/C mice (Charles River Co., Ltd., China) were used to detect cell tumorigenesis. The animals were housed in an environment of 24°C±1°C and 60% ± 5% relative humidity. In total, 24 mice were grouped using the random number table method, with 6 mice in each group. Briefly, 5 × 10^6^ CT26-wt cells were resuspended in 200 *μ*L of PBS and injected into the armpit area of the mice. Twenty-eight days after transplantation, the mice were sacrificed and tumors were collected for weighing. The animals were euthanized when the diameter of the tumor was >2 cm (not involved in this experiment). Protocols involving animals were approved by the ethics committee of the People's Hospital of Guangxi Zhuang Autonomous Region.

### 2.14. Statistical Analysis

All experiments were independently performed in triplicate. Data were expressed as mean ± SD. Statistical analysis was performed using one-way analysis of variance and Tukey's multiple comparison tests (GraphPad Prism version 7.0). The *t*-test was used to analyze the differences between the two groups. Survival analysis was performed using the logrank test. A *P* value of < 0.05 was considered statistically significant.

## 3. Results

### 3.1. LINC00174 Is Overexpressed in Colon Cancer and Associated with a Poor Prognosis

**Figure 1:**
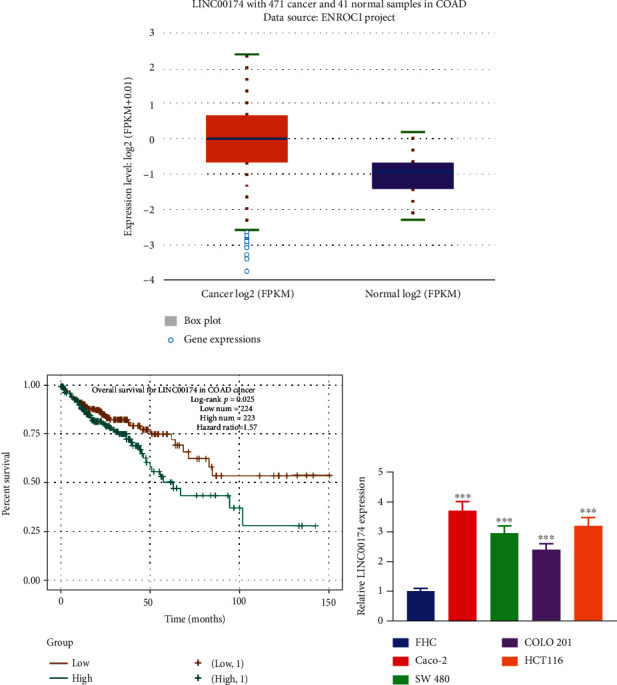
LINC00174 is overexpressed in colon cancer and associated with poor prognosis. (a) LINC00174 expression in colon cancer tissues in The Cancer Genome Atlas database. (b) The relationship between different LINC00174 levels and the survival rate of patients with colon cancer. (c) LINC0017 expression in human colonic epithelial cells and colon cancer cell lines. ^∗∗∗^*P* < 0.001 vs. FHC.

To preliminarily analyze LINC00174 expression in colon cancer, LINC00174 expression in colon cancer tissues in TCGA database was obtained for analysis. LINC00174 expression was significantly increased in colon cancer tissues (Figure 1(a)). Patients with high LINC00174 expression had a significantly decreased survival rate (*P* = 0.025) (Figure 1(b)). Compared with normal colon epithelial cells, LINC00174 expression was significantly upregulated in colon cancer cell lines (Figure 1(c)). This suggests that LINC00174 plays a positive role in colon cancer. Caco-2 cells with relatively high LINC00174 expression and COLO201 cells with relatively low LINC00174 expression were used in the subsequent experiments.

### 3.2. Silencing or Overexpression of LINC00174 Affects the Growth, Migration, and Invasion of Caco-2 Cells

**Figure 2:**
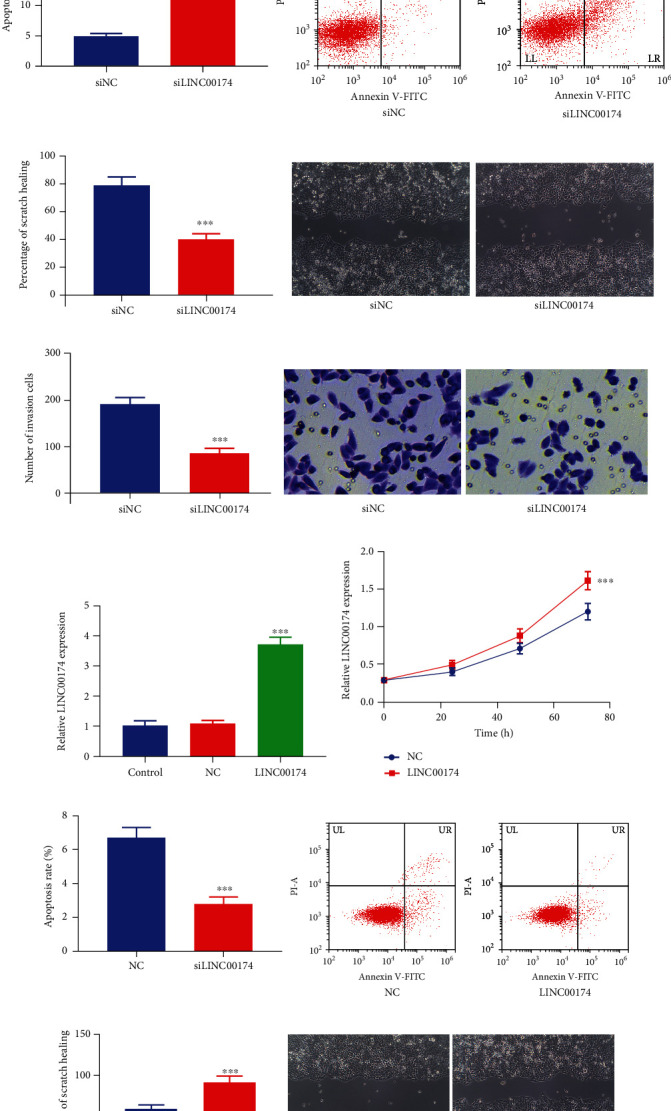
Silencing or overexpression of LINC00174 affects the growth, migration, and invasion of Caco-2 cells. (a) Construction of LINC00174-silenced Caco-2 cells. (b) Effects of LINC00174 silencing on proliferation ability. (c) Effects of LINC00174 silencing on the apoptosis of Caco-2 cells. (d) Effects LINC00174 silencing on cell migration. (e) Effects of LINC00174 silencing on the invasion of Caco-2 cells. ^∗∗∗^*P* < 0.001 vs. siNC. (f) Construction of LINC00174-overexpressing Caco-2 cells. (g) Effects of LINC00174 overexpression on proliferation ability. (h) Effects of LINC00174 overexpression on the apoptosis of COLO201 cells. (i) Effects of LINC00174 overexpression on cell migration. (j) Effects of LINC00174 expression on the invasion of COLO201 cells. ^∗∗∗^*P* < 0.001 vs. NC.

To determine the effects of LINC00174 on colon cancer cells, Caco-2 was used as a model cell line for LINC00174 silencing.  Figure 2(a) illustrates the transfection results. Cell viability was significantly decreased after LINC00174 silencing (Figure 2(b)). After LINC00174 inhibition, the apoptosis rate increased from approximately 5% to approximately 20% (Figure 2(c)). The level of scratch healing was significantly decreased in the siLINC00174 group after 24 h (Figure 2(d)). Furthermore, the number of cells that invaded the lower chamber of the Transwell device was lower in the siLINC00174 group than in the siNC group (Figure 2(e)).

COLO201 cells with relatively low LINC00174 expression were used to overexpress LINC00174 (Figure 2(f)). When LINC00174 expression increased in the cells, the proliferation ability significantly increased, whereas the apoptosis rate decreased (Figures 2(g) and 2(h)). Increasing LINC00174 expression increased the scratch healing rate from 60% to approximately 90% after 24 h (Figure 2(i)). The number of cells that invaded the lower chamber of the Transwell device was higher in the LINC00174 group than in the NC group (Figure 2(j)).

### 3.3. Silencing or Overexpression of LINC00174 Affects Inflammation in Caco-2 Cells

**Figure 3:**
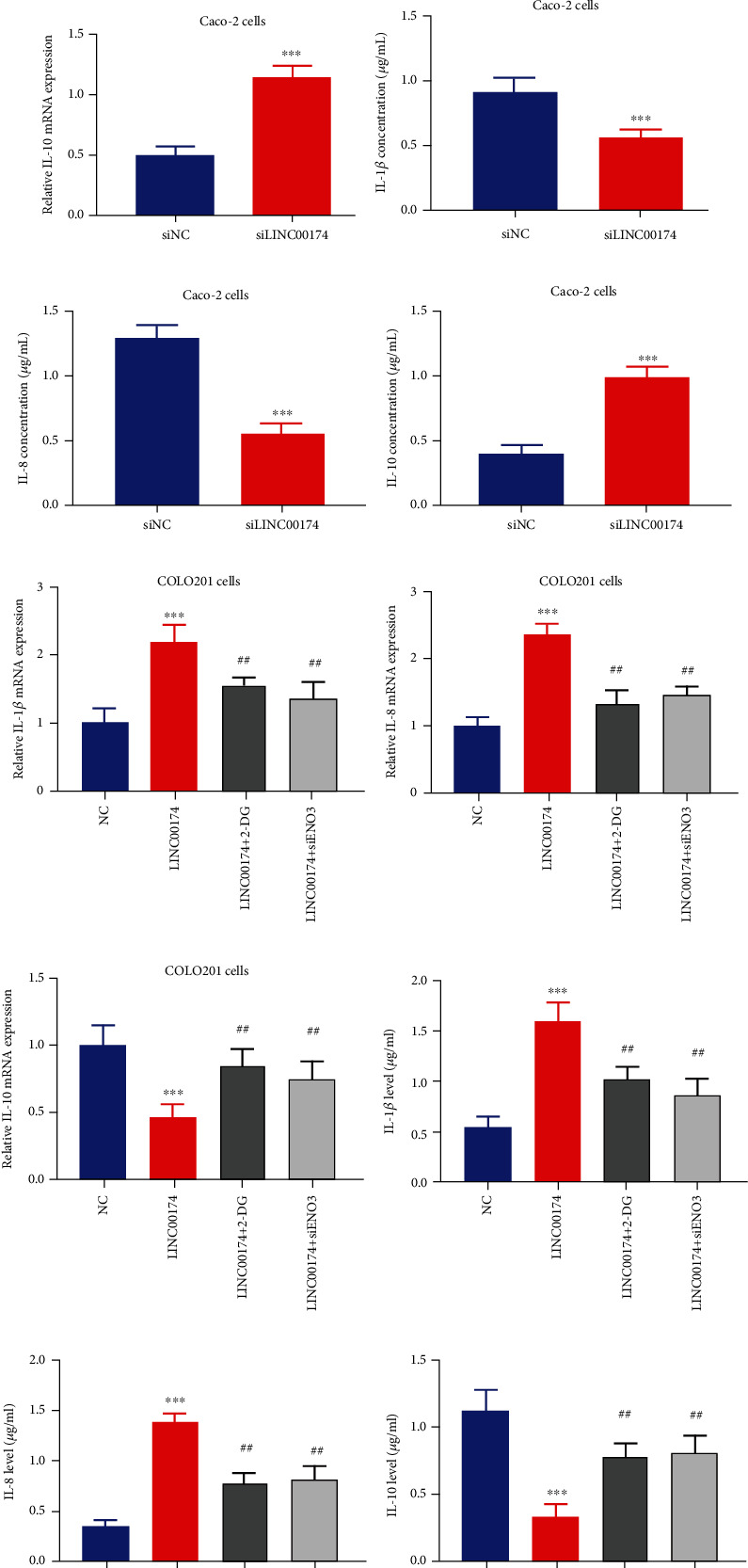
Silencing or overexpression of LINC00174 affects inflammation in colon cancer cells. (a–c) Effects of LINC00174 silencing on the transcription of the proinflammatory genes IL-1*β* and IL-8 and the anti-inflammatory gene IL-10 in Caco-2 cells. ^∗∗∗^*P* < 0.001 vs. siNC. (d–f) Effects of LINC00174 silencing on the expression of the proinflammatory genes IL-1*β* and IL-8 and the anti-inflammatory gene IL-10 in Caco-2 cells. ^∗∗∗^*P* < 0.001 vs. siNC. (g–i) Effects of LINC00174 overexpression or 2-deoxy-d-glucose (2-DG) (a glycolytic inhibitor) or ENO3 ablation on the mRNA expression of the proinflammatory genes IL-1*β* and IL-8 and anti-inflammatory gene IL-10 in COLO201 cells. ^∗∗∗^*P* < 0.001 vs. NC and ^##^*P* < 0.05 vs. LINC00174. (j–l) Effects of LINC00174 overexpression or 2-DG or ENO3 ablation on the levels of the proinflammatory genes IL-1*β* and IL-8 and anti-inflammatory gene IL-10 in Caco-2 cells. ^∗∗∗^*P* < 0.001 vs. NC and ^##^*P* < 0.05 vs. LINC00174.

A decrease in LINC00174 expression decreased the expression of the proinflammatory factors IL-1*β* and IL-8 (Figures 3(a) and 3(b)) but increased the mRNA expression of the anti-inflammatory factor IL-10 (Figure 3(c)). These results suggest that LINC00174 silencing inhibits the malignant biological behavior of and inflammation in colon cancer cells. ELISA revealed that IL-1*β* and IL-8 levels were significantly decreased in the siLINC00174 group, whereas IL-10 levels were significantly increased in the siLINC00174 group compared with the siNC group (Figures 3(d)–3(f)). Moreover, LINC0017 overexpression promoted the mRNA expression of the proinflammatory factors IL-1*β* and IL-8 (Figures 3(g) and 3(h)) but decreased that of the anti-inflammatory factor IL-10 (Figure 3(i)). This further confirms the promoting effects of LINC00174 on colon cancer. In contrast, when LINC00174-overexpressing cells were treated with 2-DG, a glycolytic inhibitor, or ablated with ENO3 (siENO3), the effects of LINC00174 overexpression on the inflammatory factors were significantly abolished (Figures 3(g)–3(l)). Compared with the LINC00174 group, IL-1*β* and IL-8 levels were significantly decreased in the LINC00174+2-DG and LINC00174+siENO3 groups (Figures 3(g), 3(h), 3(j), and 3(k)), whereas IL-10 levels were significantly increased in these two groups (Figures 3(i) and 3(l)).

### 3.4. Construction of the LINC00174/miR-2467-3p/ENO3 Axis

**Figure 4:**
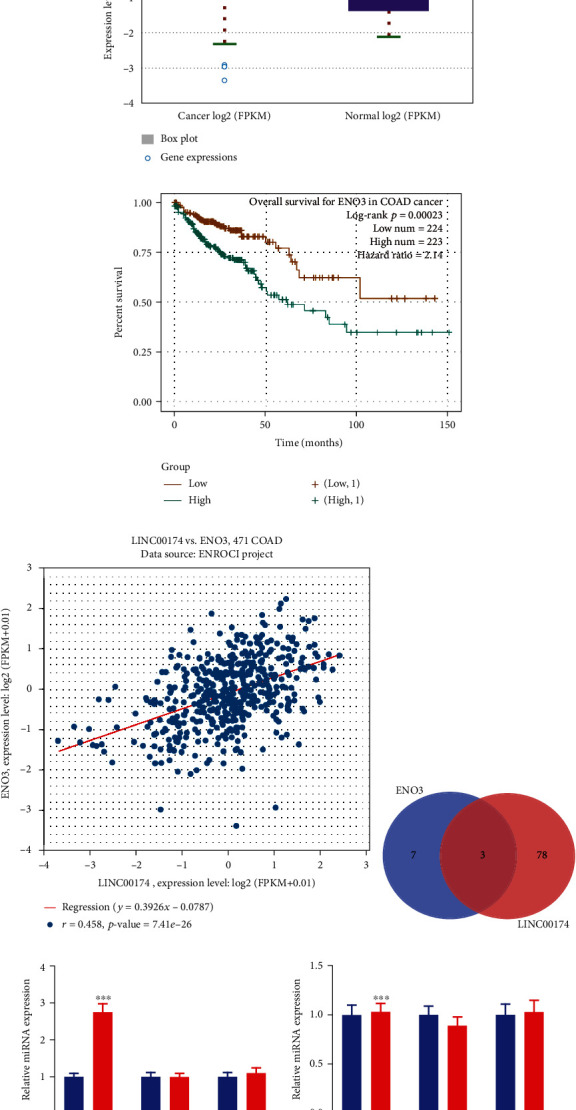
Construction of the LINC00174/miR-2467-3p/ENO3 axis. (a) ENO3 expression in colon cancer tissues in The Cancer Genome Atlas database. (b) The relationship between different ENO3 levels and the survival rate of patients with colon cancer. (c) Correlation between LINC00174 and ENO3 in colon tissues. (d) The target miRNAs of LINC00174 and ENO3; three common miRNAs were identified: miR-2467-3p, miR-3918, and miR-542-3p. (e) Effects of LINC00174 silencing on the expression of miR-2467-3p, miR-3918, and miR-542-3p in Caco-2 cells. (f) Effects of LINC00174 overexpression on the expression of miR-2467-3p, miR-3918, and miR-542-3p in COLO201 cells. ^∗∗∗^*P* < 0.001 vs. siNC or NC.

TCGA analysis revealed that ENO3 expression was increased in colon cancer tissues (Figure 4(a)). Patients with high ENO3 expression had a significantly decreased survival rate (*P* = 0.025) (Figure 4(b)). Using the colon cancer samples in TCGA database, LINC00174 was found to be positively correlated with ENO3 (*r* = 0.458, *P* < 0.001) (Figure 4(c)). The respective target miRNAs of LINC00174 and ENO3 were predicted, and three common miRNAs were identified: miR-2467-3p, miR-3918, and miR-542-3p (Figure 4(d)). To determine the differences in these three miRNAs, the effects of LINC00174 alterations on them were examined. LINC00174 silencing increased miR-2467-3p levels in Caco-2 cells; however, it had no significant effects on miR-3918 and miR-542-3p levels (Figure 4(e)). Similarly, LINC00174 overexpression suppressed miR-2467-3p levels in COLO201 cells; however, the effects on the other two miRNAs were not statistically significant (Figure 4(f)). Taken together, these results suggest that miR-2467-3p is a key miRNA in colon cancer regulation via LINC00174.

### 3.5. LINC00174 Targets miR-2467-3p to Regulate ENO3

**Figure 5:**
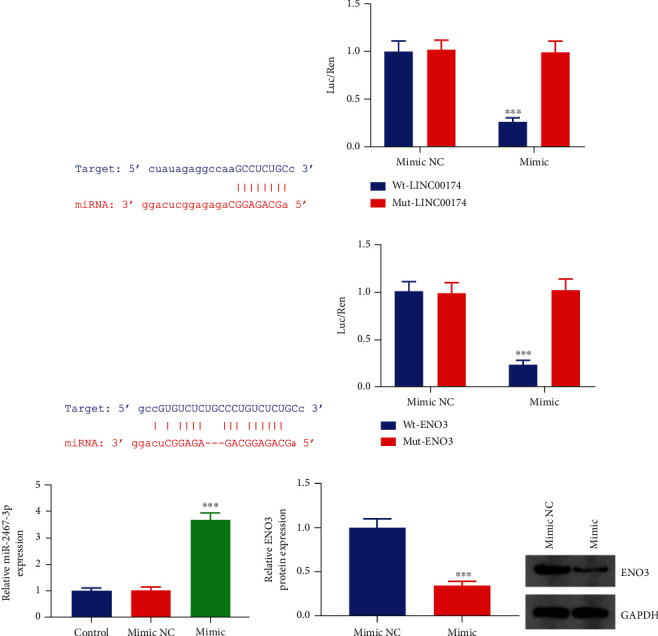
LINC00174 targets miR-2467-3p to regulate ENO3. (a) Binding sites of LINC00174 and miR-2467-3p. (b) Dual-luciferase reporter assay to verify the targeted binding between LINC00174 and miR-2467-3p. (c) Binding sites of ENO3 and miR-2467-3p. (d) Dual-luciferase reporter assay to verify the targeted binding between ENO3 and miR-2467-3p. (e) Construction of miR-2467-3p-overexpressing COLO201 cells. (f) Effects of miR-2467-3p overexpression on ENO3 protein levels in cells.

To preliminarily verify the LINC00174/miR-2467-3p/ENO3 axis, cell experiments were performed; the results demonstrated that LINC00174 bound to miR-2467-3p and that miR-2467-3p bound to the 3′-UTR of ENO3 (Figures 5(a)–5(d)). Furthermore, miR-2467-3p-overexpressing COLO201 cells were constructed (Figure 5(e)). Elevated miR-2467-3p levels inhibited ENO3 protein levels (Figure 5(f)). This suggests that LINC00174 targets miR-2467-3p to regulate ENO3.

### 3.6. LINC00174/miR-2467-3p Regulates ENO3 Expression and Colon Cell Biological Behavior and Glycolysis

**Figure 6:**
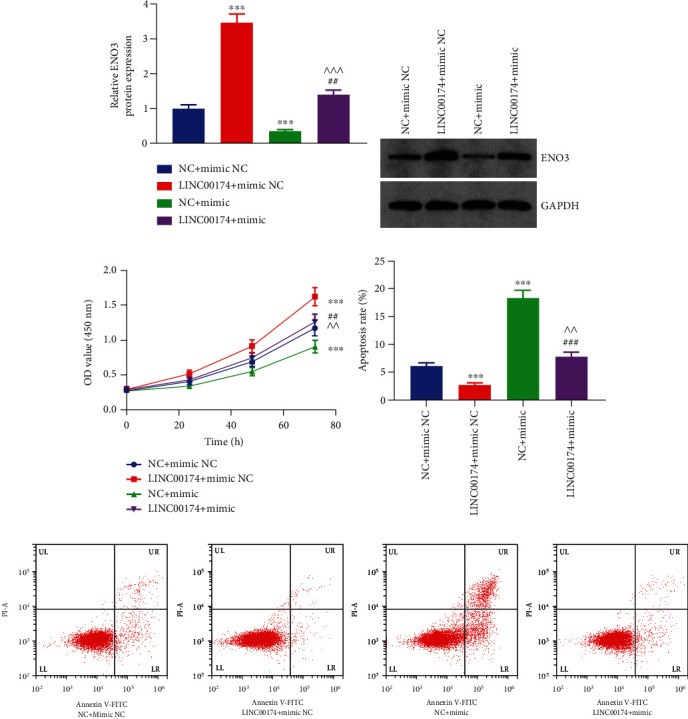
LINC00174/miR-2467-3p regulates ENO3 expression and inhibits cell proliferation. (a, b) Effects of LINC00174 and/or miR-2467-3p expression on ENO3 protein levels. (c) Effects of LINC00174 and/or miR-2467-3p overexpression on cell proliferation ability. (d) Effects of LINC00174 and/or miR-2467-3p overexpression on the apoptosis of COLO201 cells. ^∗∗∗^*P* < 0.001 vs. NC+mimic NC, ^##^*P* < 0.01 and ^###^*P* < 0.001 vs. LINC00174+mimic NC, and ^^^^*P* < 0.01 vs. NC+mimic. (e) Representative images of flow cytometry results.

To determine the LINC00174/miR-2467-3p/ENO3 axis and its effect on colon cells, COLO201 cells overexpressing LINC00174 and/or miR-2467-3p were constructed via transfection. Elevated LINC00174 levels increased ENO3 protein levels, whereas miR-2467-3p inhibited ENO3 protein levels (Figures 6(a) and 6(b)). Furthermore, LINC00174 overexpression reversed ENO3 repression via miR-2467-3p. This suggests that LINC00174 promotes ENO3 protein levels by targeting miR-2467-3p.

**Figure 7:**
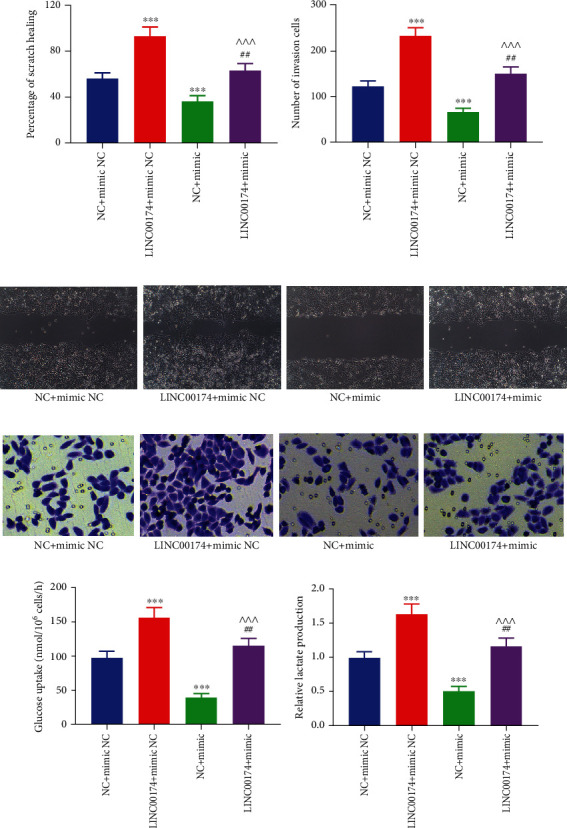
LINC00174/miR-2467-3p regulates colon cancer cell migration, invasion, and glycolysis. (a, c) Effects of LINC00174 and/or miR-2467-3p overexpression on cell migration. (b, d) Effects of LINC00174 and/or miR-2467-3p expression on the invasion of COLO201 cells. (e) Effects of LINC00174 and/or miR-2467-3p overexpression on glucose uptake. (f) Effects of LINC00174 and/or miR-2467-3p overexpression on lactate production in COLO201 cells. ^∗∗∗^*P* < 0.001 vs. NC+mimic NC, ^##^*P* < 0.01 vs. LINC00174+mimic NC, and ^^^^^*P* < 0.001 vs. NC+mimic.

Elevated miR-2467-3p levels decreased cell proliferation ability, whereas LINC00174 overexpression not only promoted cell proliferation ability but also reversed the inhibitory effects of miR-2467-3p on the cells (Figure 6(c)). Furthermore, miR-2467-3p overexpression blocked the antiapoptotic effect of LINC00174 (Figures 6(d) and 6(e)). Compared with the NC+mimic NC group, the migration and invasive abilities of the LINC00174+mimic NC group were increased. Furthermore, the migration and invasive abilities of the LINC00174+mimic group were significantly lower than those of the LINC00174+mimic NC group and significantly higher than those of the NC+mimic group (Figures 7(a)–7(d)). LINC00174 overexpression reversed the inhibitory effects of miR-2467-3p on cell motility. Taken together, the results suggest that the function of LINC00174 in regulating colon cancer is inseparable from that of miR-2467-3p.

Tumor cells absorb large amounts of glucose via glycolysis and produce lactate. Because ENO3 is involved in sugar metabolism and glycolysis, we elucidated the effects of LINC00174 and/or miR-2467-3p overexpression on glycolysis levels. Elevation of miR-2467-3p decreased glucose uptake and inhibited lactate production in COLO201 cells. However, LINC00174 overexpression not only promoted glucose uptake and increased lactate accumulation in colon cancer cells but also blocked the inhibitory effects of miR-2467-3p on glycolysis (Figures 7(e) and 7(f)). This suggests that LINC00174/miR-2467-3p not only regulates ENO3 but also regulates glycolysis.

### 3.7. LINC00174 Regulates ENO3 Protein Levels, Tumor Growth, and Inflammation by Targeting miR-2467-3p

**Figure 8:**
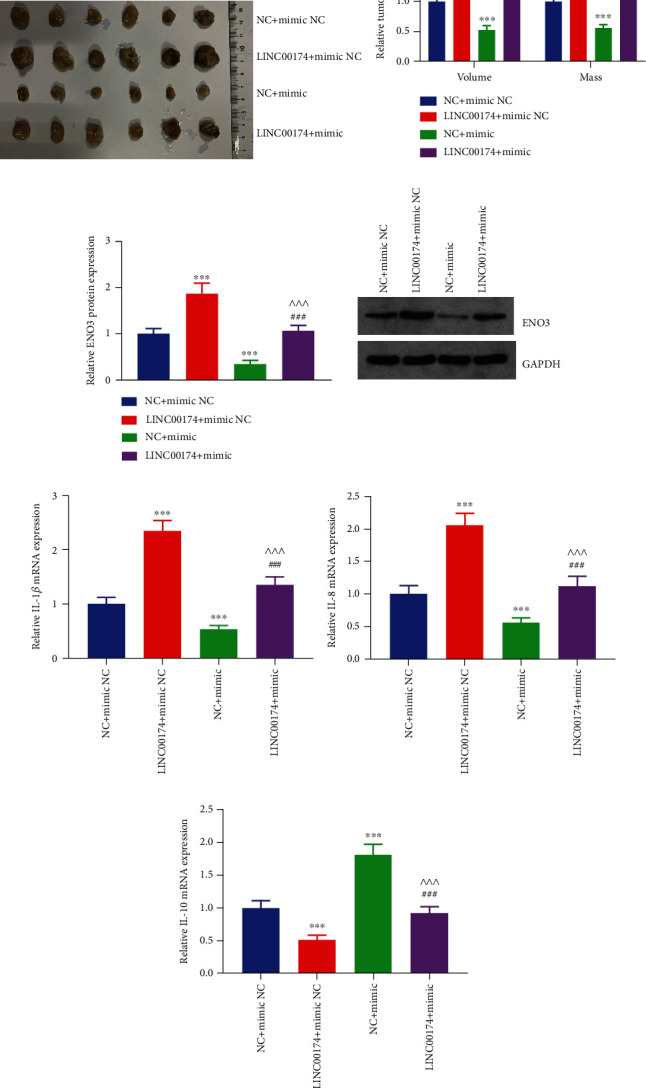
LINC00174 regulates ENO3 protein levels, tumor growth, and inflammation in tumor tissues by targeting miR-2467-3p. (a, b) Effects of LINC00174 and/or miR-2467-3p overexpression on tumor volume and mass in a tumor-bearing mice model. (c) Effects of LINC00174 and/or miR-2467-3p overexpression on ENO3 protein levels in tumor tissues. (d, e) Effects of LINC00174 and/or miR-2467-3p overexpression on the transcription of the proinflammatory genes IL-1*β* and IL-8 in tumor tissues. (f) Effects of LINC00174 and/or miR-2467-3p overexpression on the transcription of the anti-inflammatory gene IL-10. ^∗∗∗^*P* < 0.001 vs. NC+mimic NC, ^##^*P* < 0.01 and ^###^*P* < 0.001 vs. LINC00174+mimic NC, and ^^^^^*P* < 0.001 vs. NC+mimic.

To further verify the effects and mechanism of LINC00174 on colon cancer cells *in vivo*, COLO201 cells with LINC00174 and/or miR-2467-3p overexpression were used to construct a tumor-bearing mice model. LINC00174 overexpression increased tumor volume and mass by approximately 1.8-fold, whereas miR-2467-3p overexpression inhibited tumor growth to half of that observed in the NC+mimic NC group (Figures 8(a) and 8(b)). The tumor volume and mass of the mice in the LINC00174+mimic group were significantly lower than those of the mice in the LINC00174+mimic NC group and significantly higher than those of the mice in the NC+mimic group (Figures 8(a) and 8(b)). This indicates that miR-2467-3p overexpression blocks the *in vivo* promoting effects of LINC00174 on colon cancer and confirms that these effects are inseparable from those of miR-2467-3p. In addition, LINC00174 and miR-2467-3p promoted and inhibited ENO3 protein levels in vivo, respectively (Figure 8(c)). Moreover, LINC00174 overexpression reversed the inhibitory effects of ENO3 protein levels on tumor tissues via miR-2467-3p (Figure 8(c)). These findings further confirm that LINC00174 increases ENO3 protein levels by targeting miR-2467-3p *in vivo*.

We also elucidated the inflammation levels in the tumor tissues of each group and observed that the mRNA expression of IL-1*β* and IL-8 was increased but that of IL-10 decreased after LINC00174 overexpression. miR-2467-3p overexpression had opposite effects (Figures 8(d)–8(f)). In addition, the mRNA expression of IL-1*β* and IL-8 was higher in the LINC00174+mimic group than in the LINC00174+mimic NC group and lower in the NC+mimic group; however, the mRNA expression of IL-10 was lower in the LINC00174+mimic NC group and higher in the NC+mimic group compared to NC+mimic NC group (Figures 8(a)–8(f)). miR-2467-3p overexpression blocked the promoting effects of LINC00174 on inflammation, suggesting that the mechanism by which LINC00174 promotes inflammation in colon cancer is inseparable from miR-2467-3p at the *in vivo* level.

## 4. Discussion

The interaction between innate genetic risk factors and environmental carcinogenic factors is an important aspect that leads to colon cancer [30, 31]. Inflammatory bowel disease and other conditions may also lead to colon cancer, and inflammation is a crucial factor involved in colon cancer progression [32, 33]. In addition to surgery and chemotherapy, new immunotherapies are constantly being used in clinical settings; however, the prognosis of patients with colon cancer remains poor [34, 35]. Furthermore, most patients lose the best opportunity for surgery at the time of diagnosis and exhibit infiltration and metastasis [36, 37]. Therefore, it is important to analyze the mechanism underlying colon cancer progression. Glycolysis and inflammation have a close interrelationship in cancer cells. In these cells, glycolysis is often upregulated so as to facilitate their rapid growth and proliferation [38]. This phenomenon is commonly called the Warburg effect. In addition, glycolysis can lead to the production of reactive oxygen species, which can cause cellular damage and trigger inflammatory responses [39]. The chronic activation of the inflammatory pathways in cancer cells can lead to the recruitment of immune cells and release of cytokines and chemokines, thereby promoting tumor growth and metastasis. Furthermore, inflammation in the tumor microenvironment can induce glycolysis in neighboring cells, resulting in a positive feedback loop that fuels cancer progression [40]. Therefore, glycolysis and inflammation are mutually reinforcing processes driving cancer development and progression [41]. Nevertheless, the relationship between glycolysis and inflammation in cancer cells is complex and multifaceted. While glycolysis is important for cancer cell survival, it also induces inflammatory responses that contribute to tumor progression [42]. Therefore, identifying strategies that target both glycolysis and inflammation may be effective in improving cancer therapy.

lncRNAs are recently discovered novel tumor diagnostic and therapeutic targets. For example, DNAJC3-AS1 [43], EGOT [44], and LINC00261 [45] are considered clinical markers of colon cancer and are involved in regulating cell proliferation and invasion. LINC00174 (ENSG00000179406) is overexpressed in liver cancer [17], glioblastoma [46], and thymic epithelial tumors [47] and exerts a cancer-promoting function. Recent studies have reported that LINC00174 is associated with the malignant pathological features of patients with colorectal cancer and that it promotes tumor progression and metastasis [48, 49]. However, the effect and mechanism of LINC00174 on the biological behavior of and inflammation in colon cancer cells remain unclear. To this end, preliminary analysis of the colon cancer samples in TCGA database revealed that LINC00174 is overexpressed and associated with a lower survival rate. Furthermore, *in vitro* experiments using colon cancer cell lines revealed that LINC00174 overexpression promotes colon cancer cell proliferation, migration, invasion, and inflammation and inhibits apoptosis. These findings suggest that LINC00174 plays tumor- and inflammation-promoting roles in colon cancer and is a biomarker for colon cancer.

Next, to analyze the mechanism by which LINC00174 promotes colon cancer, TCGA database was used to identify ENO3, which is positively correlated with LINC00174 in colon cancer tissues. Similar to LINC00174, ENO3 was also overexpressed in colon cancer and related to a poor prognosis. Thereafter, the respective target miRNAs of LINC00174 and ENO3 were predicted, and three common miRNAs were identified: miR-2467-3p, miR-3918, and miR-542-3p. To distinguish these three miRNAs, we determined the effects of LINC00174 alterations on them. LINC00174 silencing increased miR-2467-3p levels in Caco-2 cells, whereas LINC00174 overexpression decreased miR-2467-3p levels in COLO201 cells. However, the effects on the other two miRNAs were not statistically significant. Using molecular cell experiments, we confirmed that miR-2467-3p binds to LINC00174 and ENO3 and that miR-2467-3p inhibits ENO3 protein levels, which are, in turn, regulated by LINC00174. A study reported that miR-2467-3p can inhibit the metastasis of colorectal cancer cells [50]. This suggests that miR-2467-3p is a key miRNA for LINC00174 in regulating colon cancer and ENO3 protein levels.

In the present study, we observed that LINC00174 can bind to miR-2467-3p, thereby inhibiting its expression. In turn, miR-2467-3p can target and downregulate ENO3 expression. Therefore, LINC00174 indirectly regulates ENO3 expression by modulating miR-2467-3p levels. We observed that LINC00174 expression was increased, leading to a decrease in miR-2467-3p levels and an increase in ENO3 expression. This was associated with altered glucose metabolism and increased proliferation in cancer cells. Therefore, LINC00174 may play a critical role in regulating glucose metabolism and cancer cell proliferation by modulating miR-2467-3p expression and ENO3 protein levels. Nevertheless, additional studies are warranted to fully understand the molecular mechanisms involved in this regulatory network and its potential as a therapeutic target for cancer. ENO3 encodes *β*-enolase and is mainly expressed in the skeletal muscle and liver. It plays an important role in both glycogen and cholesterol metabolism [51, 52]. ENO3 deficiency can lead to metabolic myopathy [53]. Furthermore, ENO3 may have a unique function in promoting glycolysis in tumor cells. To date, several sequencing-based studies have reported that ENO3 is involved in glycolysis and colorectal or colon cancer and that elevated ENO3 promotes ATP production or glycolysis under hypoxic conditions [27, 54]. Furthermore, genome analysis using the Gene Expression Omnibus database revealed that ENO3 is involved in glycolysis regulation; LASSO Cox analysis revealed that ENO is associated with prognosis [55]. Glycolysis also regulates the biological behavior of colon cancer cells [56, 57]. In the present study, we observed that miR-2467-3p targets ENO3 expression, inhibits the malignant biological behavior of and glycolysis in colon cancer cells, and alleviates the cancer-promoting effects of LINC00174. Moreover, LINC00174 promotes glycolysis and blocks the inhibitory effects of miR-2467-3p on colon cancer cells and glycolysis. In addition, the regulatory effects of LINC00174 on tumor growth and ENO3 protein levels via miR-2467-3p were confirmed via animal experiments. In animal models, elevated LINC00174 expression promoted the mRNA expression of IL-1*β* and IL-8 but inhibited IL-10 expression. Furthermore, miR-2467-3p overexpression blocked the promoting effects of LINC00174 on inflammation. This further suggests that in colon cancer, the increase in LINC00174 expression promotes ENO3 protein levels at the posttranscriptional level by targeting miR-2467-3p, which, in turn, promotes glycolysis and inflammation, thereby promoting colon cancer progression. Because colon cancer cells require high glucose levels to sustain their rapid growth, they adapt themselves by promoting glycolysis via the Warburg effect. The complex regulation among LINC00174, miR-2467-3p, and ENO3 can be critical for cancer cell survival and proliferation, and interfering with this regulatory network can be a promising approach to developing effective cancer therapies.

## 5. Conclusion

LINC00174 can promote colon cancer cell glycolysis, inflammation, proliferation, migration, and invasion and inhibit apoptosis. The cancer-promoting mechanism of LINC00174 is related to targeting miR-2467-3p to promote ENO3 protein levels. Nevertheless, the function and mechanism of LINC00174 in colon cancer should be further confirmed at the clinical level. Furthermore, the mechanism by which LINC00174 promotes glycolysis should be confirmed *in vivo*.

## Data Availability

The datasets generated during and/or analyzed during the current study are available from the corresponding authors on reasonable request.
